# A 27-kg mucinous cystadenoma of the ovary presenting with deep vein thrombosis

**DOI:** 10.4274/tjod.93206

**Published:** 2016-03-10

**Authors:** Esra Nur Tola, Evrim Erdemoğlu, Yakup Yalçın, Filiz Alkaya Solmaz, Ebru Erdemoğlu

**Affiliations:** 1 Süleyman Demirel University Faculty of Medicine, Department of Gynecology and Obstetrics, Isparta, Turkey; 2 Süleyman Demirel University Faculty of Medicine, Department of Gynecologic Oncology, Isparta, Turkey; 3 Süleyman Demirel University Faculty of Medicine, Department of Anestesiology and Reanimation, Isparta, Turkey; 4 Şifa Hospital, Clinic of Gynecology and Obstetrics, Isparta, Turkey

**Keywords:** Abdominal enlargement, deep vein trombosis, giant ovarian tumour, ovarian mucinous cystadenoma

## Abstract

Giant ovarian adenomas are rarely observed today because of early diagnosis and treatment. Mucinous cystadenomas is a kind of tumor that mostly causes the ovary to enlarge. Theu can present with various and non-specific clinical manifestations such as deep vein thrombosis. The primary symptoms of giant ovarian tumors are abdominal enlargement and distension. Therefore, making the correct preoperative diagnosis is sometimes difficult. The appropriate treatment must include oncologic procedures and a multidisciplinary approach to minimalize complications and save the patient’s life.

Herein, we report a woman aged 53 years with a 27-kg ovarian mucinous cystadenoma that presented as a left popliteal vein thrombosis.

## INTRODUCTION

Giant ovarian tumours are now a rare condition because of early diagnosis and treatment. Mucinous cystadenoma (MCA), a tumor that causes ovaries to enlarge, accounts for about 15% of all ovarian neoplasms^(1,2)^. About 80% of mucinous tumors are benign and are common usually between the third and sixth decade of life^(2,3)^. Mucinous tumors are usually unilateral and can exceed 30 cm in diameter^(4)^. Although prognosis is very good, the clinical signs and symptoms of ovarian masses are generally nonspecific. Therefore, making the correct preoperative diagnosis is sometimes difficult, and early management may be necessary to save the patient’s life. The purpose of this case was to report a 27-kg ovarian MCA that presented as deep vein thrombosis (DVT).

## CASE REPORT

**Figure 1:**
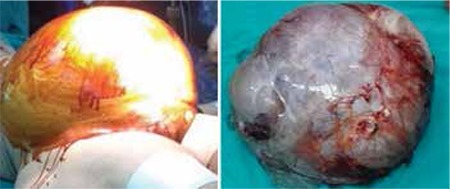
Massive distension of abdomen and giant mucinous ovarian cystadenoma

The patient was a woman aged 53 years with an abdominal mass that caused left popliteal and femoral vein thrombosis. She claimed stepwise enlargement of the abdomen and initially thought she had put on weight. However, when swelling progressed in her left leg, she was admitted to an internal medicine clinic. Her condition was diagnosed as DVT and she was referred to us upon finding an abdominal mass in her abdominal ultrasound (USG) examination. Abdominal enlargement and distension were observed, and abdominal USG revealed a 40×50 cm abdominopelvic mass, probably originating from her left ovary, with multiple septations and solid areas. Tumor markers (CA 19-9, CA 125) were within the normal range. A malignant ovarian tumor was initially suspected because of the size, and a positron-emission tomography-computerized tomography (PET-CT) was performed. The PET-CT revealed an enormous heterogenic mass with low metabolic activity in the left ovary, a 5×5 cm mass in the right ovary, and bilateral urinary stasis. A laparotomy with midline incision revealed a smooth-surfaced mass that filled the entire abdomen originating from the left ovary and confirmed the 5×5 cm mass in the right ovary (Figure 1). A detailed pelvic examination was performed because of the risk of borderline potential, and it did not indicate metastases. There was minimal free fluid in the pouch of Douglas. The patient had hypotension and bradycardia, and we quickly decided to operate in the lateral position to prevent hemodynamic derangements. The ovarian masses were sent for frozen sectioning. A hysterectomy and bilateral salpingo-oophorectomy was performed because of the giant size of the cystic mass, the presence of a contralateral ovarian mass, and the patient’s age. The cystic mass weighed 27 kg. The masses were diagnosed as benign ovarian MCA.

## DISCUSSION

Giant ovarian cystadenomas are rarely observed today because of improved imaging techniques(5). Further, most ovarian cyst adenomas are epithelial cystadenomas, of which 25% are mucinous^(6)^. MCAs mainly occur during middle age, as in our case, and they are rare among adolescents. There was, however, a report of an ovarian MCA in a premenarchal girl^(6)^. MCAs are usually unilateral, and are bilateral in 10% of cases (as in our case)^(1,7)^. MCAs can reach large sizes even though they do not have the potential for malignancy^(1,3,8)^. The largest reported adnexal cyst was 148 kg^(9)^. The mass in our patient weighed 27 kg.

In general, MCAs can be found incidentally in routine USG screenings; abdominal enlargement and distension are major symptoms. The most frequent complications are torsion, pressure to the adjacent structures like the viscera, which accounts for urinary stasis and rupture, and spilling of tumor into the peritoneum, known as pseudomyxoma peritonei^(7)^. In literature, there is no information about an ovarian mass presenting as DVT due to femoral vein pressure.

Ultrasonographic examination of ovarian cysts can exclude malignancy. In most cases, USG examination can reveal a complex adnexal mass, as in our case. When there is a diagnostic doubt, a CT or magnetic resonance imaging scan is performed. In our case, PET-CT scan was performed because of a suspicion of malignancy. Although tumor markers can identify cyst characterization in most instances, they are more important in monitoring postoperative relapse^(6)^.

Large ovarian cysts traditionally require laparotomy because of their size^(10)^. Cystectomy cannot be performed because of the absence of normal tissue; adnexectomy is the classic treatment. If there is a suspicion of malignancy, a laparotomy with oncologic procedures is recommended^(7)^. Although some studies suggest laparoscopy, there are still debates because of tumor size limitations^(10)^. We preferred laparotomy owing to the tumor dimension and suspicion of malignancy^(2,5)^.

Giant ovarian cysts have significant morbidity if not managed properly. A multidisciplinary approach must be taken preoperatively. Giant ovarian cysts can cause hemodynamic and pulmonary complications; therefore, central venous pressure monitorization is recommended during operations. The lateral decubitus position is preferred during the operation.

## CONCLUSION

*MCAs can present as different clinical conditions such as DVT. The appropriate treatment must include oncologic procedures, and a multidisciplinary approach must be undertaken to minimalize complications*.
